# Mannose-binding lectin-deficient genotypes as a risk factor of pneumococcal meningitis in infants

**DOI:** 10.1371/journal.pone.0178377

**Published:** 2017-05-31

**Authors:** Carles Bautista-Rodriguez, Cristian Launes, Iolanda Jordan, Maria Andres, Maria Teresa Arias, Francisco Lozano, Juan Jose Garcia-Garcia, Carmen Muñoz-Almagro

**Affiliations:** 1 Pediatrics Department, University Hospital Sant Joan de Deu, Barcelona, Spain; 2 CIBER de Epidemiologia y Salud Publica (CIBERESP), Instituto de Salud Carlos III, Madrid, Spain; 3 Pediatric Intensive Care Department, University Hospital Sant Joan de Deu, Barcelona, Spain; 4 School of Medicine, University of Barcelona, Barcelona, Spain; 5 Molecular Microbiology Department, University Hospital Sant Joan de Deu, Barcelona, Spain; 6 Department of Immunology, Centre de Diagnostic Biomedic, Hospital Clinic of Barcelona, Barcelona, Spain; 7 Immunoreceptors of the Innate and Adaptive Systems, Institut Investigacions Biomediques August Pi i Sunyer (IDIBAPS), Barcelona, Spain; 8 School of Medicine, Universitat Internacional de Catalunya, Barcelona, Spain; Public Health England, UNITED KINGDOM

## Abstract

**Objectives:**

The objective of this study was to evaluate to evaluate the role of mannose-binding-lectin deficient genotypes in pneumococcal meningitis (PM) in children.

**Methods:**

We performed a 16-year retrospective study (January 2001 to March 2016) including patients ≤ 18 years with PM. Variables including attack rate of pneumococcal serotype (high or low invasive capacity) and *MBL2* genotypes associated with low serum MBL levels were recorded.

**Results:**

Forty-eight patients were included in the study. Median age was 18.5 months and 17/48 episodes (35.4%) occurred in children ≤ 12 months old. Serotypes with high-invasive disease potential were identified in 15/48 episodes (31.2%). *MBL2* deficient genotypes accounted for 18.8% (9/48). Children ≤ 12 months old had a 7-fold risk (95% CI: 1.6–29.9; p < 0.01) of having a *MBL2* deficient genotype in comparison to those > 12 months old. A sub-analysis of patients by age group revealed significant proportions of carriers of *MBL2* deficient genotypes among those ≤ 12 months old with PM caused by opportunistic serotypes (54.5%), admitted to the PICU (Pediatric Intensive Care Unit) (46.7%) and of White ethnicity (35.7%). These proportions were significantly higher than in older children (all p<0.05).

**Conclusions:**

Our results suggest that differences in MBL2 genotype in children ≤12 months old affects susceptibility to PM, and it may have an important role in the episodes caused by non-high invasive disease potential serotypes.

## Introduction

*Streptococcus pneumoniae* remains a serious health problem and a leading cause of life-threatening invasive infection in young children. More than 14 million cases of pneumococcal disease occur annually in children aged < 5 years with a mortality rate over 5% [1]. *S*. *pneumoniae* is the main cause of bacterial meningitis in children beyond the neonatal period [1]. The morbility and case-fatality ratio for pneumococcal meningitis is high. The average global pneumococcal meningitis case-fatality ratio has been estimated to be 59%” [1].

Children are often colonized with *S*. *pneumoniae* in the nasopharynx. Capsular polysaccharides are the main virulence factors of pneumococci. There are more than 95 different capsular types (serotypes) but not all have the same ability to invade and cause disease. Some *S*. *pneumoniae* serotypes are considered to have a low-invasive capacity to cause disease and are often found in carriers. They are common colonizers specially in children < 2 years and have more temporal opportunity for invasion in this age range. They are also called “opportunistic serotypes” [2,3]. In contrast, serotypes with a high-invasive capacity to cause disease are seldom detected in carriers but they are an important cause of invasive disease specially in older children [4,5]. Serotypes are classified according to the studies of Brueggemann [4] Sleeman [5] and del Amo [6]: 1, 4, 5, 7F, 8, 9V, 12F, 14, 18C and 19A are considered to have a high-attack rate or highly-invasive serotypes whereas the remainder are considered as low-attack rate or non-highly-invasive or opportunistic serotypes. These highly-invasive serotypes are generally included in the pneumococcal vaccines (PCV13 or PPSV23).

The complex interaction between impaired host immune factors and virulence determinants of the pneumococcus plays a role in the development of invasive pneumococcal disease (IPD) [7]. Components of the host innate immune response are important first-line defense factors against infections in young children since the adaptive response is still developing and often altered with reduced antibody response and of shorter persistence [8]. Mannose-binding lectin (MBL) is a proinflammatory protein of the innate immune system involved in complement activation via the lectin pathway and it provides immediate host defense against infection [9]. Current knowledge holds that MBL serum levels largely depend on the *MBL2* genotype [10–12]. MBL has the capacity of activating the complement cascade independent of antibody response [9] and its plasma concentration is genetically determined [13,14]. *S*. *pneumoniae* shows low to intermediate MBL binding capacity and MBL-deficiency has been associated with a 5-fold increased risk of death due to pneumococcal disease [15].

Evidence supports MBL-deficiency as a risk factor for developing IPD [9], higher risk of developing severe respiratory complications in neonates and young children [16], higher bacterial loads during meningococcal sepsis in young children [17], and increased risk for higher frequency and duration of infectious complications in children with malignancies [18]. However, its role in host defense to pneumococcus remains a matter of debate. It has been considered to predispose to IPD for some authors, while others have discarded it [9,19,20].

The objective of the present study is to evaluate PM susceptibility in pediatric patients considering the major virulence factor of pneumococci, capsular type and the genetic variation within the *MBL2* gene as an important host immune factor. This information could be useful for designing preventive strategies on children based on previous analysis of host-pathogen interactions.

## Materials and methods

### Setting, population and design

The Pediatric Pneumococcal Surveillance Study Group based at Hospital Sant Joan de Deu (Barcelona) has been prospectively collecting epidemiological, clinical and analytical data from children with IPD since 1988. In this retrospective observational study we included patients ≤ 18 years of age with community acquired PM from our database between 1 January 2001 and 31 March 2016 (conjugated vaccine era). Children were classified in <12 months of age and >12 months of age. PM was defined as an *S*. *pneumoniae* positive cerebrospinal fluid (CSF) culture and/or *S*. *pneumoniae* DNA detection in CSF.

### Microbiologic methods

Pneumococcal isolates were identified and serotyped using standard methods in the molecular microbiology department at Hospital Sant Joan de Deu. DNA detection of pneumolysin (*ply*) and *LytA* gene by Real-Time PCR (*polymerase chain reaction*) in CSF was performed according to previously reported assays [21–24].

Serotypes were classified according to published scientific assays [4–6]: 1, 4, 5, 7F, 8, 9V, 12F, 14, 18C and 19A were considered as high-attack rate or highly-invasive serotypes whereas the remainder were considered as low-attack rate or non-highly-invasive or opportunistic serotypes.

### *MBL2* polymorphism analysis

DNA extraction, amplification and genotyping of *MBL2* were carried out as previously described [25]. Six single nucleotide polymorphisms (SNPs) in the 5’-flanking/promoter region (-550 G/C -221 C/G, 4 C/T) and the exon 1 (codon 52 CGT/TGT, codon 54 GGC/GAC and codon 57 GGA/GAA) of the *MBL2* gene were analyzed using a PCR and Sequence-Based Typing (SBT) technique. The D, B and C variants at codons 52, 54 and 57, respectively, are major determinants of low serum MBL levels [13]. As previously described by Ali YM et al [20], these variants are collectively named O, while A indicates the wild-type variant at all those codons. The SNPs at positions –551 [H/L], −221 (X/L) and +4 (P/Q) also influence serum MBL levels in individuals with the wild-type A variant. However, the functional effects of H/L and P/Q SNPs appear to be minor compared to L/X, X being the allele associated with lower MBL expression. Accordingly, MBL serum concentrations can be divided into the following 3 genotype groups: high (A/A, A/XA), intermediate (XA/XA, A/O) and low (XA/O, O/O).

### Data collection and exclusion criteria

Clinical information of patients was extracted from medical records and recorded on a standardized case report form that included demographics, clinical presentation, laboratory results, neuroimaging findings, management, complications and neurological sequelae. Children with functional deficit of classical or alternative pathways of complement activation were excluded from the study, as well as patients with immunocompromised conditions (human immunodeficiency virus infection, immunoglobulin deficits), cystic fibrosis, bronchiectasis or cerebrospinal leak.

### Statistical analysis

Statistical analysis was performed using Statistical Package for Social Sciences Software (IBM SPSS® 22 version). Descriptive statistics was used to characterize the study population. The χ^2^ test and Fisher exact test were used to compare categorical variables. Continuous non-normally distributed variables were described in terms of median value with interquartile range (IQR, 25^th^ percentile - 75^th^ percentile) and compared using Mann-Whitney *U* test. Risk associations are presented as relative risk with 95% confidence intervals. A 2-tailed *P* value <0.05 was considered statistically significant.

### Data confidentiality and ethical aspects

All information collected was treated as confidential in strict observance of legislation. The study was approved by the Ethics Committee of Hospital Sant Joan de Deu (Permit Number: CEIC PIC 98–13) and conducted according to the principles expressed in the Declaration of Helsinki. All subjects and their legal representatives included in the study received detailed information about the aims of the study prior to recruitment. Written informed consent was obtained from all patients/legal representatives.

## Results

### Characteristics of the study population

A total of 821 IPD episodes occurred during the study period; 104 PM episodes (12.7%) were identified among 93 children (29/93, 27.9% were <12 months). 12/104 episodes (11.5%) were excluded due to exclusion criteria [cerebrospinal leak]; 33/104 episodes (31.7%) refused to participate in the study. 8/104 children (7.7%) died due to the infection. All of the patients who died but one were under 2 years of age. They were mainly male (75%). The pneumococcal strain was serotyped in 5 of the patients (4/5 were opportunistic serotypes). Among those that were serotyped, all but one are included in the 13v pneumococcal vaccine. No MBL genotypes were available for those patients. Thus, the final study sample comprised 48 episodes with PM.

There was a predominance of males (29/48; 60.4%). The predominant ethnic group was White (43/48; 89.6%). Median age was 18.5 months (IQR, 7.2–54.2) and 17/48 episodes (35.4%) occurred in children ≤ 12 months old. There was a median LOS of 17 days (IQR, 13–24). In 43/48 episodes (89.6%) admission to the Pediatric Intensive Care Unit (PICU) was required. Only 15/48 patients (31.2%) had previously been completely vaccinated (10 with PCV7 and 5 with PCV13); 31/48 (64.6%) suffered sequelae (such as motor impairment, seizures, psychomotor retardation, hearing loss or loss of vision). Table 1 shows demographic, clinical and microbiological variables of patients according to age category.

**Table 1:** Demographic, clinical and microbiological variables of patients according to age category.

Characteristics	≤12 months	>12 months	Total	p-value
**Subjects**	17	31	48	
**Male**	12 (70.6)	17 (54.8)	29 (60.4)	0.29
**White Ethnicity**	14 (82.3)	29 (93.5)	43 (89.6)	0.22
**PICU admission**	15 (88.2)	28 (90.3)	43 (89.6)	0.82
**LOS days [IC95%]**	20.8 (15.4–26.1)	25.2 (9.4–41.2)		0.75
**Sequelae**	8 (47.1)	23 (74.2)	31 (64.6)	0.06
**Non-vaccinated according to age**	11 (64.7)	22 (71.0)	33 (68.8)	0.65
**PM caused by low invasive serotypes**	11 (64.7)	22 (71.0)	33 (68.7)	0.65
**PCV13 serotypes**	10 (58.8)	20 (64.5)	30 (62.5)	0.70
***MBL2* deficient genotypes**	7 (41.2)	2 (6.4)	9 (18.7)	**0.03**

PICU, pediatric intensive care unit; LOS, length of stay; PM, pneumococcal meningitis; PCV13, 13-valent pneumococcal vaccine; MBL, mannose-binding-lectin

Significant values in bold numbers

### Diagnosis and serotyping

Among all episodes, 35.4% (17/48) were confirmed only by PCR, 20.8% (10/48) by culture and 43.8% (21/48) by both PCR and culture. No patient was positive for any other bacteria other than *S*.*pneumoniae* in CSF or blood cultures or PCR analysis. Capsular serotypes were available for all PM cases. Serotypes 19A, 19F and 3 were the most frequent serotypes and accounted for about 31.2% of the infections (19A, n = 5/48; 19F, n = 5/48 and 3, n = 5/48). Serotypes with high-invasive capacity were identified in 15/48 episodes (31.2%). The currently used PCV13 vaccine would have covered 62.5% of serotypes that caused PM (30 out of 48 episodes). Genotype of *MBL2* gene was available for all 48 patients. Deficient-MBL associated genotypes accounted for 18.8% (9/48). Table 2 provides an overview of haplotype frequencies.

**Table 2:** Overview of haplotypes frequencies in children with pneumococcal meningitis.

Promoter Exon	YY	YX	XX
**AA**	A/A n = 15 (31.3%)	A/XA n = 10 (20.8%)	XA/XA n = 4 (8.3%)
**AO**	A/O n = 10 (20.8%)	XA/O n = 4 (8.3%)	
**OO**	O/O n = 5 (10.4%)		

MBL serum concentrations were divided into the following 3 groups: normal (A/A, A/XA), intermediate (XA/XA, A/O) and deficient (XA/O, O/O). Mannose-binding-lectin (MBL) protein expression: white: normal; light grey: intermediate; dark grey: defective.

Median age in patients with *MBL2* deficient genotypes was 8.0 months (IQR 4.0–15.5) compared to 44.5 months (IQR 10.0–67.0) in patients with *MBL2* non-deficient genotypes (p = 0.01). Children ≤ 12 months old had a 7-fold risk of having a low-MBL genotype in comparison to those > 12 months old (Relative-Risk = 7.00 (95% CI: 1.6–29.9; p < 0.01). A sub-analysis of patients by age category (considering children’s age at the cut-off value of 12 months old) revealed significant proportions of carriers of *MBL2* deficient genotypes among those ≤ 12 months old with PM caused by opportunistic serotypes (54.5%, 6/11), admitted to the PICU (46.7%, 7/15) and of white ethnicity (35.7%, 5/14). These proportions were significantly higher than in older children (p < 0.05) (Table 3).

**Table 3:** Variable frequencies according to age group and *MBL2* genotype.

	Age group ≤12 months		Age group > 12 months		
Variables	Total number of patients	Patients with *MBL2* deficient genotypes	Total number of patients	Patients with *MBL2* deficient genotypes	p-value*
**Serotype invasiveness**					
Opportunistic serotype	11	6 (54.5%)	22	1 (4.55%)	**< 0.01**
High invasiveness	6	1 (16.6%)	9	1 (11.1%)	0.76
**White ethnicity**	14	5 (35.7%)	29	2 (6.9%)	**0.02**
**Clinical course with sequelae**	8	3 (37.5%)	23	2 (8.7%)	0.06
**PICU admission**	15	7 (46.7%)	28	2 (7.1%)	**<0.01**

Significant values in bold numbers.

MBL, mannose-binding-lectin; PICU, pediatric intensive care unit.

* Proportions between groups were compared using Pearson Chi-square test or Fisher's exact test when appropriate

*MBL2* deficient genotypes were not found to be significantly associated with sex, ethnicity, serotype invasiveness, PICU admission, vaccination status or clinical course variables. Data regarding associations between variables and *MBL2* genotypes are presented in Table 4.

**Table 4:** Variable frequencies according to *MBL2* genotype.

Variables	Patients with *MBL2* deficient genotypes	Patients with *MBL2* non-deficient genotypes	*p* value
Age [months], IQR	**8.0 (4.0–15.5)**	**44.5 (10.0–67.0)**	**0.01**
Female	5 (55.6)	14 (35.9)	0.27
White ethnicity	7 (77.8)	36 (92.3)	0.19
PICU admission	9 (100)	34 (87.2)	0.25
LOS days (SD)	26.56 (±24.79)	20.28 (±13.27)	0.29
Clinical Course with sequelae	5 (55.6)	26 (66.7)	0.53
Non-high invasiveness / opportunistic serotype	7 (77.8)	26 (66.7)	0.51

Data are presented as *n* (%). IQR (interquartile range)

Significant values in bold numbers

PICU, pediatric intensive care unit; LOS, length of stay; SD, standard deviation

## Discussion

This study highlights the relationship between genetically determined MBL deficiency and the increased risk of pneumococcal meningitis in children younger than 12 months old, especially by serotypes with low invasive capacity or opportunistic serotypes.

Susceptibility to IPD is higher in younger children [1,2]. Children under 2 years of age rely on their innate immune response to overcome infections, which is critical to avoid the spread of nasopharyngeal colonizing organisms into sterile human body sites [8]. MBL is a circulating protein of the innate immune system and its deficiency has been reported to predispose to IPD [9]. A previous study of our group suggested that a genetically determined low-MBL production could be associated with IPD when disease occurs in children under 2 years of age [24]. In the present study, we also found a significantly higher proportion of *MBL2* deficient genotypes among children ≤ 12 months with PM. The frequency of *MBL2* deficient genotypes was significantly higher in children ≤12 months of age in comparison to older patients (41.1% vs 6.2%), whereas the overall *MBL2* deficient genotypes prevalence in adults from our setting is around 10–15% [26]. These data suggest that children with *MBL2* deficient genotypes are at higher risk of PM in their first year of life. This is in agreement with Brouwer et al report on association of low-producing *MBL2* genotypes and increased risk of PM [27].

On the other hand, IPD in children is often caused by serotypes with low invasive disease potential [3], as we report in our series (68.8% of the PM were caused by opportunistic serotypes). Considering only the episodes caused by opportunistic serotypes, we observed that the proportion of individuals with *MBL2* deficient genotypes was higher in children ≤ 12 months old than in older ones. Nevertheless, this finding was not observed in those children with PM caused by high-invasive disease potential. In our opinion, this suggests that low invasive capacity serotypes may have more opportunities to cause invasive disease in young children with *MBL2* deficient genotypes as their immunity relies specifically in the innate immune system. In our previous study, the frequency of *MBL2* deficient genotypes was especially high in younger children with IPD caused by opportunistic serotypes too [24]. At this point, it is important to underline that 13-valent conjugated vaccine would have protected against the main serotypes causing PM in our series, achieving protective antibody titers with prompt vaccination schedules [28].

A potential limitation in this study could be the assumption that patients were healthy prior to meningitis. Some of the children might have unidentified immunodeficiency or other chronic diseases that had not been diagnosed at the time of meningitis. However we have tried to lower this bias through the exclusion criteria and long follow-up. Secondly, the role of other protein receptors such as ficolins, pentraxins or salivary agglutinin/gp340/DMBTI known to be involved in the innate immune system response to *S*. *pneumonia* has not been studied [29]. Third, we did not analyze other factors that may be involved in the set from colonization to disease and, in particular, the role of co-infection with respiratory viruses [30,31]. Fourth, if the sample size was larger the difference between the conditions compared could be measured with greater confidence. However, this is harder to achieve in pediatric studies.

In conclusion, the importance of MBL in susceptibility to infection is largely discussed and remains controversial [9,19,20,32], but our results suggest that genetic variation in the *MBL2* gene could affect the susceptibility to PM in children ≤12 months old, and it may have a more important role in the episodes caused by non-high invasive disease potential serotypes. This association suggests that the risk of IPD cannot only be assessed by clinical, epidemiological or microbiological factors but also by the immune characteristics of the youngest hosts. In this regard, the conceptual framework known as PIRO (predisposition, infection characteristics, host response and organ dysfunction) [33] could be a tool not only for understanding the physiopathology of meningitis but also for helping to better the prognosis and be able to design appropriate interventions and evaluate the impact that they represent. We believe that knowing what factors are most important for morbidity and mortality in our patients may help to stratify risk and improve the use of health resources. Children with the risk factors noted above may be more aggressively monitored and treated and preventive strategies, such as early vaccination, strongly recommended.

## Supporting information

S1 Table Supporting data for the manuscript results. (XLSX)
